# A Comparative Multi-Scale Study on the Regeneration Mechanisms of a Bio-Based and a Petroleum-Based Asphalt Rejuvenator

**DOI:** 10.3390/ma19163547

**Published:** 2026-08-21

**Authors:** Xiying Yang, Wencai Zhang, Xiaogang Guo, Mingqian Zhai

**Affiliations:** 1 School of Transportation Engineering, Shanxi Vocational University of Engineering Science and Technology, Jinzhong 030619, China; yangxiying@sxgkd.edu.cn (X.Y.); zhaimingqian@sxgkd.edu.cn (M.Z.); 2 Larson Transportation Institute, Pennsylvania State University, University Park, PA 16802, USA; gangxo@outlook.com

**Keywords:** aged asphalt, asphalt rejuvenator, bio-based, petroleum-based, regeneration mechanism

## Abstract

Growing demand for pavement maintenance promotes reclaimed asphalt pavement (RAP) recycling, yet multi-scale comparative research on rejuvenators remains limited. This study compared a commercial bio-based Rejuvenator A and a petroleum-based Rejuvenator B to reveal how chemical compositions control asphalt regeneration. Following JTG 3410-2025 standards, asphalt binders underwent extended RTFOT aging (75, 112.5, and 150 min) and were externally blended with rejuvenators at 5%, 7.5%, and 10% by binder mass. A multi-scale framework combining macro tests, interfacial characterization, SARA fraction analysis, ATR-FTIR, and molecular dynamics (MD) simulations was established. Results indicated that the two rejuvenators acted via different mechanisms. Rejuvenator B contained 31.64% saturates and restored aged asphalt mainly through physical dilution and softening. This rejuvenator formed 0–80 hydrogen bonds dominated by weak C–H···O interactions, and the corresponding rejuvenated asphalt yielded a CII of 0.293. It was effective for 75 min-aged binders yet produced more oxidation products after re-aging. Conversely, Rejuvenator A, characterized by 68.10% aromatics and 20.17% resins, exhibited a 3–5° lower equilibrium contact angle, and a roughly 13% shorter relative penetration time than Rejuvenator B. Its network of 150–250 hydrogen bonds effectively disrupted asphaltene aggregates, reducing the CII to 0.180. Consequently, Rejuvenator A successfully restored 112.5 min- and 150 min-aged asphalt with significantly lower secondary oxidation. These performance differences were governed by aromatic and polar fractions rather than feedstock origins. While quantitative findings are sample-specific, the established framework provides theoretical references for engineering rejuvenator selection and high-performance regenerant design.

## 1. Introduction

With the arrival of the large-scale maintenance era of road infrastructure, the resource recycling of Reclaimed Asphalt Pavement (RAP) has become a key factor in promoting the green and low-carbon development of the civil engineering industry. Asphalt rejuvenators are core materials for increasing RAP content and ensuring the performance of recycled pavements. Within a circular economy framework, they enable the repeated reuse of pavement materials, reducing virgin bitumen consumption and construction waste [1]. However, significant differences exist in the action mechanisms and effects of different types of rejuvenators on aged asphalt [2]. At present, petroleum-based rejuvenators are mainly prepared from petroleum refining by-products and have been widely applied in civil engineering. Bio-based rejuvenators are produced using plant oils as raw materials and have gradually attracted research attention in recent years [3]. However, the compositional causes behind their distinct regeneration efficiencies and internal action mechanisms remain unclear, which creates obstacles to rational on-site selection of rejuvenators for RAP recycling projects.

In recent years, the optimization of asphalt rejuvenators has become a prominent research focus, yet most existing literature predominantly evaluates the macroscopic performance recovery of a single type of rejuvenator rather than systematically comparing their underlying mechanisms. For instance, researchers have extensively explored the physical and rheological restoration capabilities of various organic and biological oils. Studies incorporating waste cooking oil (WCO) and other organic oils into RAP mixtures have demonstrated measurable improvements in macroscopic physical properties through a series of laboratory tests [4]. Similarly, the application of microalgae oil has been shown to favorably modify the chemical, physical, and mechanical properties of aged 70/100 asphalt binders, revealing its potential as a sustainable rejuvenator [5]. Beyond bio-oils, chemical agents such as dilauryl thiodipropionate (DLTDP) have also been investigated through dynamic shear rheology to validate their feasibility in RAP applications [6]. While these studies successfully verify the macroscopic effectiveness of various rejuvenators, they often treat the regeneration process as a whole, lacking a comprehensive investigation into the influence of micro-components on the structural reorganization of aged asphalt.

To elucidate the intrinsic mechanisms behind the performance recovery of rejuvenated asphalt, microscopic characterization has been widely adopted to investigate microstructural evolution. Techniques including saturates, aromatics, resins and asphaltenes (SARA) fractionation, gel permeation chromatography (GPC), and attenuated total reflection Fourier-transform infrared (ATR-FTIR) spectroscopy have proven effective in tracking the evolution of chemical fractions, molecular agglomeration, and functional groups [7,8,9]. Despite these advancements, there is a lack of systematic comparison regarding how bio-based and petroleum-based rejuvenators differentially regulate these components.

Conventional macroscopic and microscopic test means are incapable of molecular-scale observation of intermolecular interactions between asphalt binders and rejuvenators, thereby restricting the systematic elucidation of the intrinsic regeneration mechanism of aged asphalt. In this regard, molecular dynamics (MD) simulation effectively compensates for this technical defect. Existing simulations build asphalt molecular models based on classic SARA molecular frameworks and calculate indexes such as the diffusion coefficient and molecular aggregation morphology to analyze rejuvenator penetration behavior [10,11,12]. However, few studies have built corresponding correlations between microscopic molecular simulation results and macroscopic road performance.

From what has been discussed above, the existing evaluation system of asphalt rejuvenators is fragmented. Macroscopic performance tests lack microstructural interpretation, microscopic detection ignores the feedback of pavement macroscopic performance, and molecular simulation is disconnected from real interfacial diffusion behaviors. Most studies neglect the mesoscopic interfacial transition indexes, including contact angle, surface tension, adhesion work, and diffusion kinetics, that link molecular composition and bulk rheology.

To fill the above research gaps, this paper selects one typical bio-based rejuvenator (A) and one typical petroleum-based rejuvenator (B), two widely-used commercial engineering materials with distinct polar-aromatic compositions for mechanism comparison. Gradient-aged asphalt binders with three relative thermo-oxidative levels were prepared via extended RTFOT. Macroscopic conventional performance and secondary aging resistance were tested first to capture apparent performance differences. Then, mesoscopic contact angle and surface tension tests were conducted to analyze interfacial diffusion and compatibility, which act as the superficial cause of property gaps. After that, microscale SARA, GPC, and ATR-FTIR tests were carried out to reveal component and functional group differences. Finally, MD simulation based on the COMPASS II force field was adopted to quantify hydrogen bond numbers and Coulomb potential energy, so as to clarify the essential molecular interaction mechanism behind all multi-scale discrepancies. In summary, this paper aims to establish a full-chain multi-scale analytical framework covering macroscopic properties, mesoscopic interfacial behaviors, microscopic components, molecular structures, and intermolecular forces, so as to comparatively elucidate the action mechanisms of rejuvenators A and B from surface phenomena to intrinsic molecular essence.

It should be emphasized that only two representative rejuvenators are selected for discussion in this work, and the relevant conclusions and laws cannot be generalized to all bio-based or petroleum-based rejuvenators. This work provides targeted theoretical reference for the development and application of similar rejuvenator materials in RAP recycling engineering.

## 2. Materials and Methods

### 2.1. Raw Materials

#### 2.1.1. Base Asphalt

Donghai penetration grade 90 base asphalt was selected as the virgin binder, which was supplied by Sinopec Maoming Petrochemical Company (Maoming, China). Its technical specifications, measured according to the Technical Specifications for Construction of Highway Asphalt Pavements (JTG F40-2004) [13], are summarized in Table 1.

#### 2.1.2. Rejuvenators

Two rejuvenators with distinct supplier-claimed origins were evaluated: Rejuvenator A (bio-based) and Rejuvenator B (petroleum-based), which were obtained from Shanxi Xiyuefa Road Construction and Maintenance Group Co., Ltd. (Taiyuan, China). Their fundamental physical properties are presented in Table 2 [14].

To quantitatively characterize their chemical and molecular features, SARA fractionation, ATR-FTIR, and gel-permeation chromatography (GPC) tests were performed in this work. SARA analysis reflects the bulk compositional distribution including aromatic content, ATR-FTIR reveals polar functional-group information, and GPC provides auxiliary evidence regarding molecular weight distribution differences between the two rejuvenators, although it cannot identify specific functional groups or elemental composition.

It should be noted that high-precision molecular-level identification tests, including GC-MS, NMR, and elemental analysis, were not conducted under current experimental conditions. Detailed fine-scale molecular component characterization is suggested for follow-up research.

#### 2.1.3. Other Materials

All chemical reagents used for SARA fractionation and GPC analysis, including n-hexane, toluene, dichloromethane, and methanol, were of chromatographic purity and purchased from Sinopharm Chemical Reagent Co., Ltd. (Shanghai, China).

### 2.2. Sample Preparation

#### 2.2.1. Aged Asphalt

Following the JTG 3410-2025 standard [15], the Rolling Thin Film Oven Test (RTFOT) was carried out at a constant temperature of 163 °C to produce aged asphalt samples. Referring to pre-experimental data of asphalt property evolution, three heating durations of 75 min, 112.5 min, and 150 min were adopted to generate asphalt binders with different relative thermo-oxidative levels, referred to as 75 min-aged, 112.5 min-aged, and 150 min-aged asphalt binders.

It must be clarified that the extended RTFOT method in this work is a simplified aging scheme for laboratory research, which differs from the standard RTFOT combined with Pressure Aging Vessel (PAV) or UV/thermal cycling and cannot fully replicate the long-term aging behavior of asphalt in actual road service.

#### 2.2.2. Rejuvenated Asphalt

The aged asphalt binders were heated to 140 °C, and rejuvenators A and B were incorporated at dosages of 5%, 7.5%, and 10% by weight (external blending). The mixtures were stirred thoroughly and subsequently conditioned in an oven at 135 °C for 30 min to ensure adequate diffusion and homogenization before testing.

### 2.3. Characterization Methods

To comprehensively evaluate the regeneration effects and underlying mechanisms of the bio-based and petroleum-based rejuvenators, a multi-scale characterization framework was adopted. This included assessments of the macroscopic physical properties, mesoscopic interfacial behavior, and microscopic chemical composition of the asphalt binders. The main equipment used for these tests is listed in Table 3.

#### 2.3.1. Conventional Performance Tests

The macroscopic recovery of rejuvenated asphalts was quantified through a series of conventional tests, including penetration, softening point, ductility, and dynamic viscosity at 60 °C. These tests were conducted in strict accordance with the Standard Test Methods of Asphalt and Asphalt Mixture for Highway Engineering (JTG 3410-2025).

#### 2.3.2. Compatibility Test

The interfacial compatibility between the rejuvenators and the aged asphalt was evaluated using the segregation test. Samples were prepared by adding 10% rejuvenator to 150 min-aged asphalt binders and processed according to JTG 3410-2025. The system uniformity was assessed by measuring the softening point difference and analyzing the spectral subtraction of the FTIR spectra between the top and bottom portions of the samples [16].

#### 2.3.3. Apparent Interfacial Transfer Rate

Contact angles were measured using the sessile drop method. The droplet profile was captured using the camera system of the contact angle goniometer, and the contact angle values at different times were calculated using the fitting method, based on data captured during the initial 60 s of contact between the rejuvenator and aged asphalt at 1 s intervals. The average value was ultimately taken as the final contact angle. Surface tension was determined by the pendant drop method, where droplet images were captured by the instrument to calculate the surface tension of the rejuvenators.

Apparent interfacial transfer rate and transfer time were determined based on surface tension, viscosity, and contact angle using Equations (1) and (2) [17]; these parameters are used for qualitative comparison of interfacial penetration performance between two rejuvenators.

(1) $$\frac{dL}{dt}=\frac{(\gamma_{S}-\gamma_{SL})R}{4\eta L}=\frac{R\gamma_{LV}\cos\theta }{4\eta L}$$

(2) $$t=\frac{2\eta L^{2}}{R(\gamma_{S}-\gamma_{SL})}=\frac{2\eta L^{2}}{R\gamma_{LV}\cos\theta }$$

where θ is the equilibrium contact angle of the rejuvenator on the aged asphalt surface (°); γ*_LV_* is the surface tension of the rejuvenator (mN/m); η is the viscosity of the rejuvenator (mm^2^/s); *L* is the capillary length on the aged asphalt surface, normalized value (dimensionless); *R* is the capillary radius on the aged asphalt surface, normalized value (dimensionless). Note: For aged asphalt prepared under the same conditions, it can be considered that their surface structures are essentially similar. To enable a relative comparison of the two rejuvenators on identical substrates, *R* and *L* are assumed to be equal in both systems (normalized to 1). Consequently, the relative interfacial transfer rate is γ*_LV_*cos (θ)/(4η), and the relative transfer time is 2η/(γ*_LV_*cos (θ)). The resulting values are used only for qualitative comparison and do not represent absolute diffusion coefficients.

The work of adhesion (*W*_a_), work of wetting (*W*_i_), and spreading coefficient (*S*) were calculated according to the Young–Dupré equation (Equations (3)–(5)) to evaluate the interaction between the rejuvenator and aged asphalt.

(3) $$W_{a}=\gamma_{LV}(\cos\theta +1)$$

(4) $$W_{i}=\gamma_{LV}\cos\theta$$

(5) $$S=\gamma_{LV}(\cos\theta -1)$$

where *W*_a_ is the work of adhesion (mN/m); *W*_i_ is the work of wetting (mN/m); *S* is the spreading coefficient (mN/m).

#### 2.3.4. Microstructural Analysis

SARA Fraction Analysis: Thin-layer chromatography coupled with a flame ionization detector (TLC-FID) was employed. Specifically, n-hexane, toluene, and a dichloromethane–methanol mixture (by volume at a ratio of 95:5) were used sequentially as developing solvents to accurately quantify the fractions of saturates, aromatics, resins, and asphaltenes.

To characterize the evolution of asphalt colloidal structure, the colloidal instability index (CII) was calculated using measured SARA mass fractions, as expressed in Equation (6) [18]:

(6) $$\mathrm{CII}=\frac{\mathrm{Asphaltenes}+\mathrm{Saturate}}{\mathrm{Aromatic}+\mathrm{Resin}}$$

Molecular Weight Analysis: Gel permeation chromatography (GPC) was employed to determine the molecular weight and distribution of the samples. Tetrahydrofuran was used as the mobile phase, with the column temperature maintained at 30 °C and a flow rate of 1.0 mL/min.

Fourier Transform Infrared Spectroscopy (FTIR) Analysis: Attenuated Total Reflectance (ATR) mode was adopted to investigate the variations in characteristic functional groups [19]. The measurements were conducted within a wavenumber range of 4000–650 cm^−1^, with a spectral resolution of 4 cm^−1^ and 32 co-added scans.

#### 2.3.5. Molecular Dynamics (MD) Simulations

Molecular dynamics (MD) simulation was performed to provide molecular-scale interpretation of the rejuvenator–aged asphalt interactions. The asphalt molecular models were constructed based on the SARA fractionation results and previously reported molecular structures. The rejuvenator molecules were built according to their compositional characteristics derived from SARA analysis. All simulations were conducted using the COMPASS II force field within Materials Studio 2020 software under the NPT ensemble at 165 °C for 40 ns [20]. The systems were considered equilibrated when the total energy and temperature stabilized over the final 10 ns of the trajectory.

To quantitatively characterize these intermolecular interactions, hydrogen-bond identification was performed using geometric criteria widely adopted in asphalt molecular-dynamics simulations [21]. Specifically, the donor–acceptor distance was set to ≤3.5 Å, and the donor–hydrogen–acceptor angle was required to be ≥120°. These criteria capture both classical hydrogen bonds (e.g., O-H···O, N-H···O) and weaker non-classical contacts such as C-H···O, which are counted as hydrogen-bond-like interactions under this MD geometric definition. Identical criteria were applied to both rejuvenator–asphalt systems to guarantee a reasonable relative comparison.

## 3. Results

### 3.1. Performance Recovery of Rejuvenated Asphalt

#### 3.1.1. Variation Characteristics of Aged Asphalt Performance and Regeneration Effect

**Figure 1:**
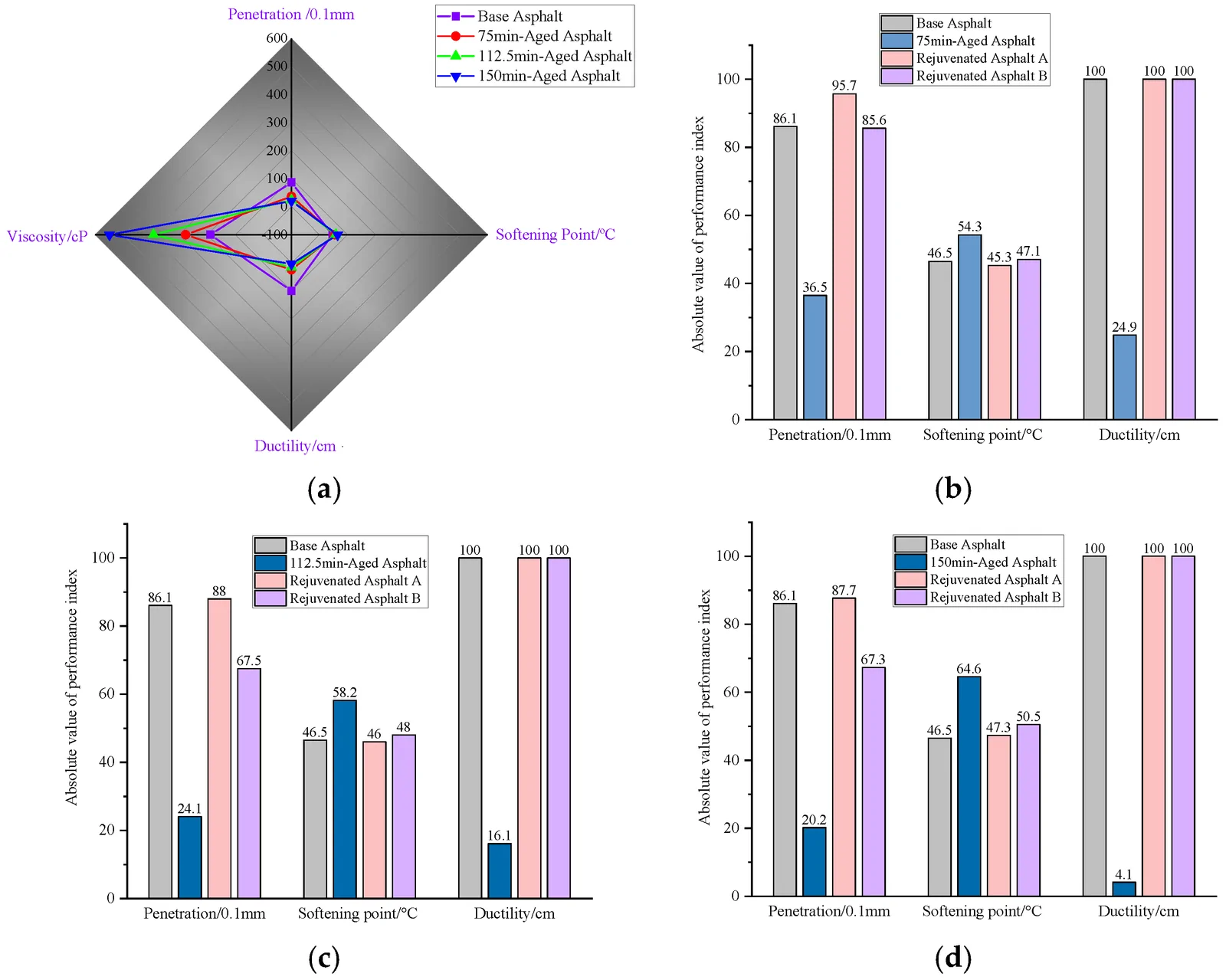
Performance comparison of asphalts with different aging degrees and rejuvenated asphalts. (**a**) Asphalts with different aging degrees. (**b**) 75 min-aged/rejuvenated asphalts. (**c**) 112.5 min-aged/rejuvenated asphalts. (**d**) 150 min-aged/rejuvenated asphalts.

Figure 1a shows that asphalt performance decays during aging, with an overall trend of decreased penetration and increased softening point. Quantitatively, for 75 min-aged asphalt binders, penetration drops by 57.6% and the softening point rises by 7.8 °C; at 112.5 min, penetration drops by 72.0% and the softening point rises by 11.7 °C; at 150 min, penetration drops by 76.5% and the softening point rises by 18.1 °C. The greater the aging degree, the more severe the performance decay.

Figure 1b–d compare the performance recovery effects of Rejuvenator A and Rejuvenator B on asphalt with different aging degrees. The data indicate that both rejuvenators A and B have obvious regeneration effects, and the penetration, softening point, and ductility of recycled asphalt are significantly recovered, but there are significant differences in recovery degree:

For 75 min-aged asphalt, the penetration of aged asphalt mixed with 5% Rejuvenator A is higher than that of base asphalt, and the softening point is lower than that of base asphalt, indicating that the regenerated asphalt becomes softer and the high-temperature stability is reduced compared with base asphalt. Therefore, 5% Rejuvenator A is excessive for 75 min-aged asphalt, and the dosage should be appropriately reduced in practical application. In contrast, the penetration and softening point of aged asphalt mixed with 5% Rejuvenator B are almost 100% recovered compared with base asphalt, showing a moderate dosage and satisfactory recovery effect.

For 112.5 min and 150 min-aged asphalt, Rejuvenator A demonstrates outstanding regeneration effects. Within the laboratory aging scope of this study, the conventional physical indices of the aged asphalt binder are essentially recovered to the level of the base asphalt. In contrast, the rejuvenation capacity exhibited by Rejuvenator B is clearly inadequate: the peak penetration recovery ratio is approximately 78%, and the softening point recovery is around 92%, which renders it challenging to restore the properties of 150 min-aged asphalt to the target range; this indicates that its repair capability for aged asphalt with severe structural damage is inferior to that of Rejuvenator A.

Overall, asphalt performance gradually deteriorates with an increase in the aging degree. Although both rejuvenators can restore the performance of aged asphalt, their restoration efficiency depends on the aging level.

#### 3.1.2. Comparative Study on Secondary Aging of Rejuvenated Asphalts

**Figure 2:**
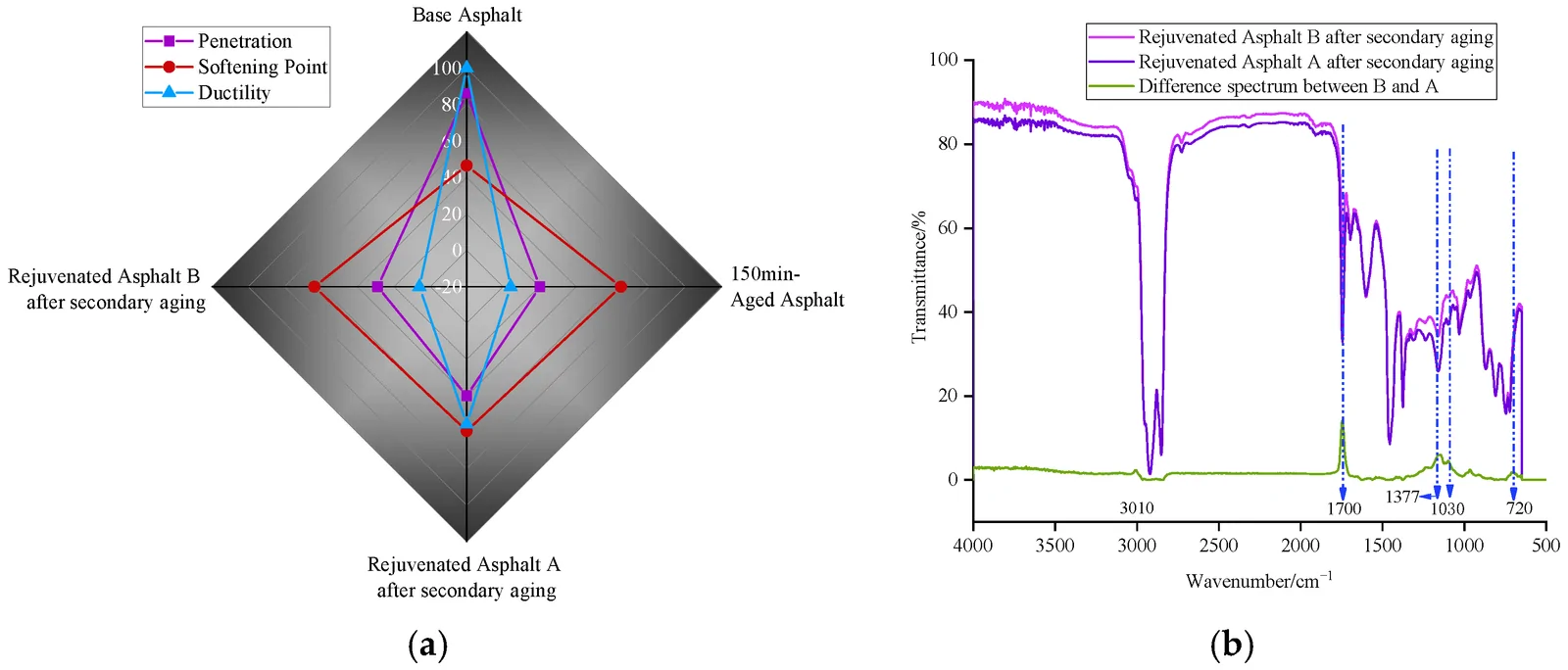
Comparison of secondary aging results of rejuvenated asphalts. (**a**) Secondary aging performance. (**b**) Secondary aging FTIR spectra.

Secondary aging tests were conducted under identical aging conditions on 150 min-rejuvenated asphalts. The results revealed the essential differences between the two rejuvenators in enhancing asphalt durability, as shown in Figure 2.

Figure 2a shows that after secondary aging, the retention rates of key indices such as penetration, softening point, and ductility for rejuvenated asphalt A were significantly higher than those for rejuvenated asphalt B. This series of data indicates that the asphalt system restored by Rejuvenator A possesses significantly better anti-aging capability than the system restored by Rejuvenator B.

Figure 2b shows that after secondary aging, the areas of the aging characteristic peaks at 1700 cm^−1^ (carbonyl) and 1030 cm^−1^ (sulfoxide) for rejuvenated asphalt B were significantly larger than those for rejuvenated asphalt A [22]. This indicates that, first, under the same secondary thermal-oxidative stress, more polar oxidation products such as carbonyl and sulfoxide groups might have formed in system B, with a more intense oxidation reaction degree and a faster chemical aging process; and second, compared to Rejuvenator B, Rejuvenator A may have a better mitigating effect on secondary aging, possessing a certain anti-aging property.

The above findings provide a crucial scientific basis for the design and selection of high-durability recycled asphalt pavement materials. Especially for pavement projects facing severe service conditions such as heavy loads and high temperatures, Rejuvenator A demonstrates significant application potential.

### 3.2. Differences in Mesoscopic Interfacial Behavior

#### 3.2.1. Analysis on Diffusion Behavior of Rejuvenator in Aged Asphalt

The diffusion behavior of rejuvenators in aged asphalt is the core kinetic mechanism for achieving effective regeneration. This part assesses the diffusion behavior of both rejuvenators within aged asphalt.

Data listed in Table 4 reveal similar diffusion patterns for the two rejuvenators across all aging gradients. More serious asphalt aging leads to a larger solid–liquid contact angle and slower interfacial transfer speed. The root cause lies in shifted surface polarity of aged asphalt binders, which weakens the wetting condition at the rejuvenator–asphalt interface [23].

**Figure 3:**
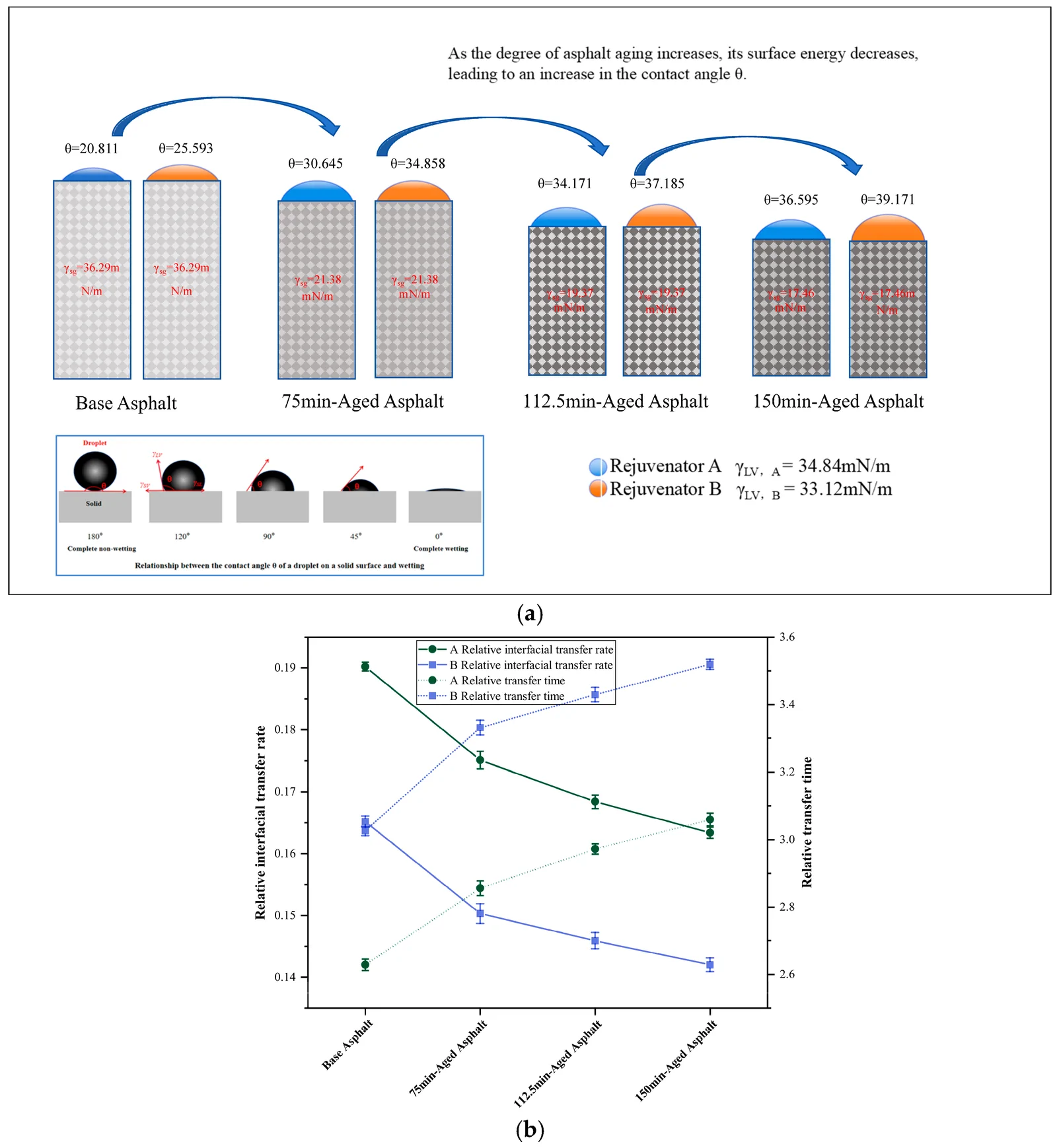
Diffusion behavior of rejuvenators and aged asphalt. (**a**) Schematic diagram of contact angle changes. (**b**) Relative interfacial transfer rate and relative transfer time.

Figure 3 reveals distinct diffusion kinetics between the two rejuvenators. Regardless of the aging level, Rejuvenator A exhibits a smaller contact angle (3–5° lower on average) and a relative interfacial transfer rate approximately 1.15 times that of Rejuvenator B. Accordingly, the relative transfer time for Rejuvenator A to reach a given penetration depth is about 13% shorter.

The primary factor contributing to the superior diffusion performance of Rejuvenator A is favorable interfacial energy compatibility with aged asphalt. Such compatibility strengthens intermolecular interactions and reduces solid–liquid interfacial tension, leading to a smaller equilibrium contact angle. This advantage overcomes the unfavorable effect induced by higher liquid–vapor surface tension and governs the improved spreading and penetration capacity.

#### 3.2.2. Interaction Between Rejuvenator and Aged Asphalt

Grounded in surface free energy (SFE) theory, the interfacial bonding mechanism between Rejuvenator agents and the aged asphalt binder is quantitatively evaluated using adhesion work (*W_a_*), spreading coefficient (*S*), and immersion work (*W_i_*) (Equations (3)–(5)). Among them, *W_a_* characterizes the interfacial bonding strength, *S* (all negative values in this study) reflects the spreading difficulty, and *W_i_* directly measures the interfacial bonding strength [24].

Data analysis in Table 5 reveals that with the intensification of asphalt aging, *W_a_* and *W_i_* of both rejuvenators decrease systematically, and the negative value of *S* increases. This indicates that asphalt aging significantly deteriorates the wetting and bonding environment of rejuvenators. Thermodynamically, the decrease in *W_a_* directly originates from the increase in the contact angle (θ) (Equation (3)), and the increase in θ means an increase in the solid–liquid interfacial tension (γ*_SL_*). This phenomenon is primarily attributed to the increased disparity in surface chemical properties between the aged asphalt and the rejuvenators, which consequently reduces their compatibility.

For asphalt samples with identical substrates, Rejuvenator A exhibits higher *W*_a_ and *W*_i_ values and a less negative spreading coefficient compared with Rejuvenator B, although the magnitude of these differences is moderate (a mere 7% difference in adhesion work). This result indicates that Rejuvenator A achieves interfacial performance comparable to or marginally superior to Rejuvenator B. This limited numerical gap can be explained by the inherent characteristics of the Dupré formula: the 3–5° difference in time-averaged dynamic contact angle provides valid advantages for wetting and diffusion, yet hardly induces prominent discrepancies in static thermodynamic parameters. Although the static interfacial difference has limited practical significance for macroscopic performance evaluation, the cumulative dynamic diffusion advantage enables Rejuvenator A to achieve superior regeneration effects on 150 min-aged asphalt. The relative interfacial transfer rate of Rejuvenator A was higher, and the relative penetration time was shortened by roughly 13%. Minor differences at the molecular interface can accumulate continuously during long-term contact and diffusion between the rejuvenator and aged asphalt, which may gradually enhance the ultimate regeneration effect. Consistently, Section 3.1 of this study demonstrates that Rejuvenator A delivers a notable recovery effect on the macroscopic properties of aged asphalt, which supports the above inference.

#### 3.2.3. Compatibility Analysis of Rejuvenators and Aged Asphalt

**Figure 4:**
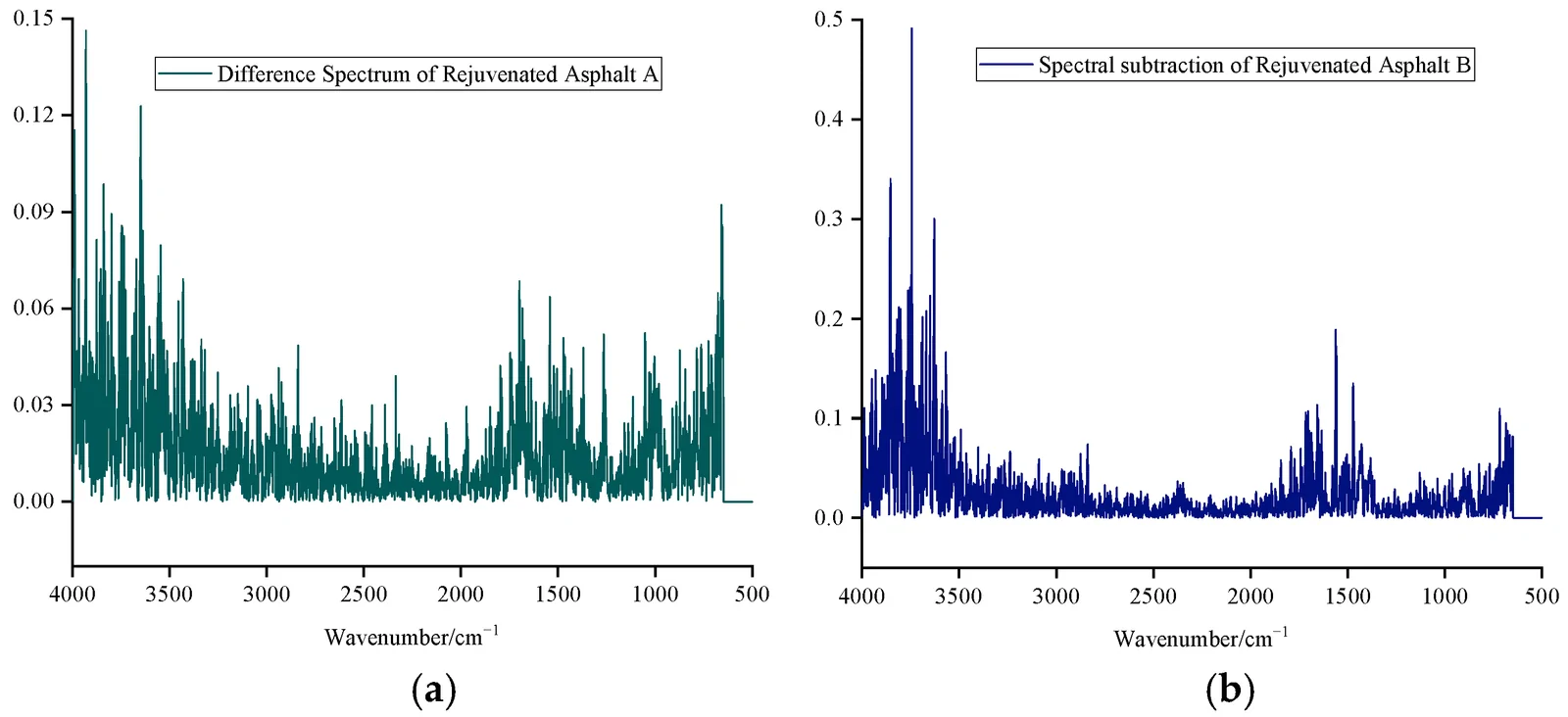
Spectral subtraction of rejuvenated asphalts from segregation test. (**a**) Rejuvenated asphalt A. (**b**) Rejuvenated asphalt B.

The interfacial compatibility between rejuvenators and aged asphalt serves as a critical factor for evaluating the homogeneity and storage stability of the regeneration system. In the present research, the segregation test was adopted to assess the compatibility of rejuvenators using two indicators—softening point difference and spectral subtraction—with the corresponding findings presented in Table 6 and Figure 4.

As shown in Table 6, the softening points of the upper sections are lower than those of the lower sections, indicating that both rejuvenators possess a lower density compared to the aged asphalt binder. As the storage duration increases, both rejuvenators exhibit a tendency to float and undergo segregation; however, the softening point differences for both samples are below the technical threshold specified in JTG F40-2005 for segregation evaluation (<2.5 °C), which verifies that both rejuvenators possess excellent compatibility with aged asphalt. Specifically, the softening point difference of rejuvenated asphalt A is recorded as 0.1 °C, while that of rejuvenated asphalt B is 0.4 °C, which confirms that the compatibility between Rejuvenator A and aged asphalt is superior to that of Rejuvenator B.

The spectral subtraction provides further validation of the aforementioned findings from a microscopic viewpoint [25]. As illustrated in Figure 4, the spectral subtraction for the rejuvenated asphalt B sample in the segregation test is 0.5, which is larger than 0.15 of recycled asphalt A. Since the sampling amount for infrared spectrum test is less than 1 g, the test results clearly reflect the system uniformity of samples taken in micro-areas. The spectral subtraction results indicate that the recycled asphalt A system has better uniformity; that is, the compatibility between Rejuvenator A and aged asphalt is better than that of Rejuvenator B, which is an important prerequisite for its efficient regeneration and stable colloidal structure formation.

### 3.3. Microstructural Chemical Composition Analysis

#### 3.3.1. SARA Analysis

To reveal the action mechanism of rejuvenators from the perspective of material microstructure composition, four-component analysis (SARA) [15] was carried out on base asphalt, 150 min-aged asphalt, recycled asphalt, and rejuvenators. The test results are illustrated in Table 7.

Following the aging of asphalt binder, its four-component composition undergoes substantial changes: the content of light fractions (including saturates and aromatics) exhibits a downward trend, whereas the asphaltene fraction rises from 9.57% to 15.91%. These findings demonstrate that throughout the asphalt aging process, the light fractions are converted into heavier components, the aggregation level of asphaltene molecules becomes more pronounced, and the colloidal structure of asphalt is disrupted, which ultimately results in the degradation of the asphalt’s macroscopic properties.

Table 7 also reveals significant differences in the four components of the two rejuvenators: Rejuvenator A is characterized by high aromatics (68.10%), high resins (20.17%), and low saturates (10.31%); Rejuvenator B shows the characteristics of high saturates (31.64%), and relatively low aromatics (52.03%) and resins (15.05%). This component difference determines the obvious distinction in the action mechanism of the two during the component recovery of aged asphalt.

Based on this, the component changes of recycled asphalt A and B were further analyzed and compared: the asphaltene content of recycled asphalt A decreases to 7.04% compared with aged asphalt, resins increase to 38.86%, and aromatics rise to 45.92%.

To quantitatively characterize the colloidal state variation, the colloidal instability index (CII) was calculated according to Equation (6). The CII values of base asphalt, aged asphalt, rejuvenated asphalt A, and rejuvenated asphalt B are 0.340, 0.427, 0.180, and 0.293, respectively. The CII rises after aging, demonstrating deteriorated colloidal stability, while blending rejuvenators reduces the CII substantially. Rejuvenated asphalt A achieves a much lower CII than rejuvenated asphalt B, confirming superior colloidal stability.

These finding suggest that the incorporation of Rejuvenator A restores the colloidal structure of aged asphalt. Specifically, its elevated aromatic fraction dissolves the asphaltene aggregates generated during aging. Concurrently, the increased resin content adsorbs onto the asphaltene surfaces to assemble micelles with asphaltene cores. Through peptization, these micelles disperse within the medium composed of aromatics and saturates, ultimately reconstructing a stable colloidal structure [26]. In contrast, recycled asphalt B has a high content of saturates (14.84%) and aromatics (46.80%) as dispersion media, and a lower resin content (30.52%) than recycled asphalt A. This indicates that Rejuvenator B rejuvenates aged asphalt mainly via light component supplementation and physical dilution, and exhibits weaker peptization capacity to break asphaltene aggregates compared with Rejuvenator A.

The differences between the two rejuvenators in components and action paths lead to different macroscopic performance and long-term durability of recycled asphalt, and also provide direct evidence for understanding the regeneration mechanism from a microscopic perspective.

#### 3.3.2. Molecular Weight Analysis

**Figure 5:**
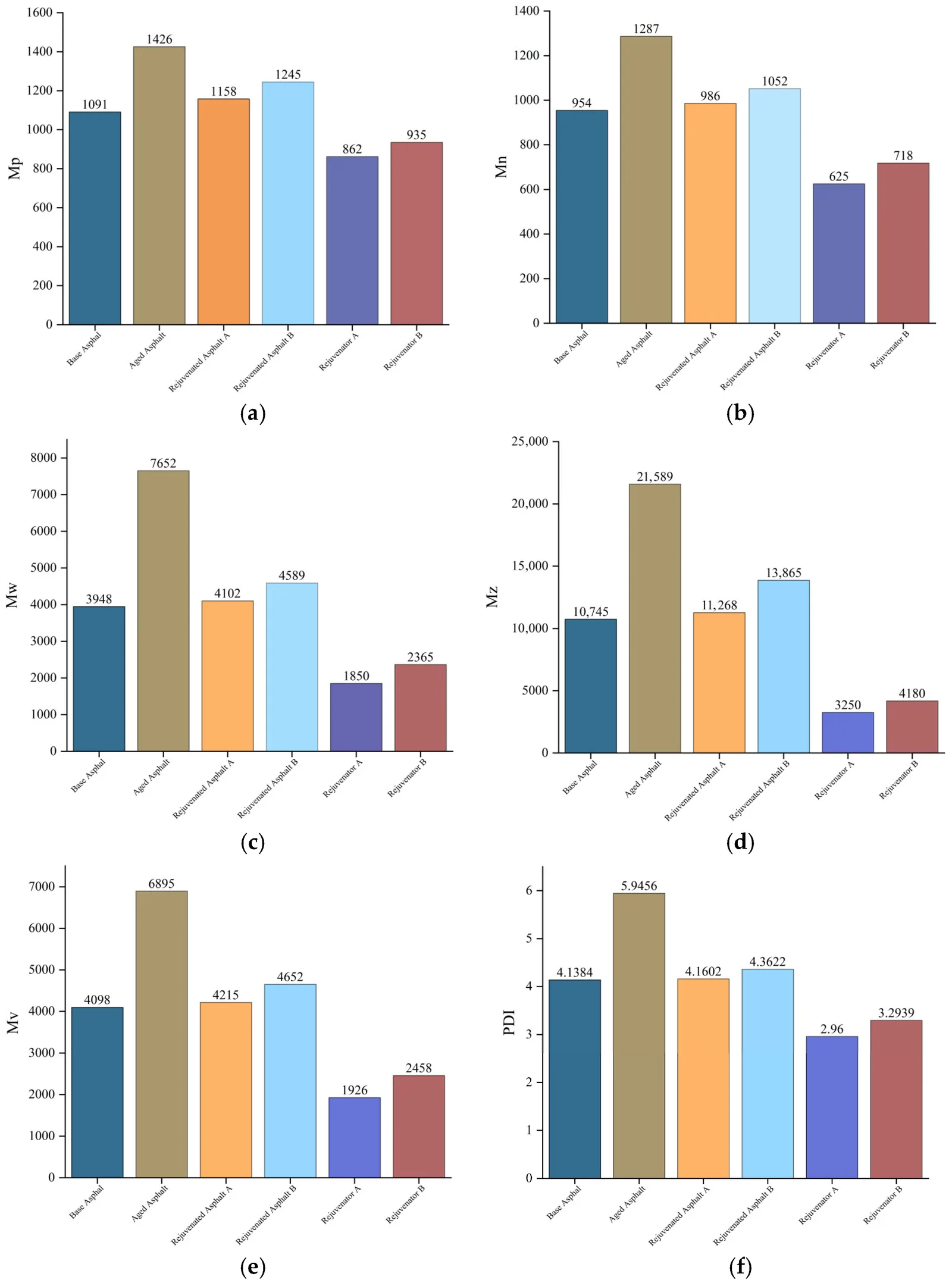
Molecular weight test results. (**a**) M_P_. (**b**) M_n_. (**c**) M_W_. (**d**) M_Z_. (**e**) M_V_. (**f**) PDI. Notes: M_p_—Peak Molecular Weight; M_n_—Number-Average Molecular Weight; M_w_—Weight-Average Molecular Weight; M_z_—Z-Average Molecular Weight; M_v_—Viscosity-Average Molecular Weight; PDI—Polydispersity Index (PDI = M_w_/M_n_), which reflects the width of molecular weight distribution (the smaller the PD, the more uniform the molecular weight distribution).

To elucidate the working mechanism of rejuvenators at the molecular level, gel permeation chromatography (GPC) was employed to characterize the molecular weight distribution of virgin asphalt [27], aged asphalt, and recycled asphalt, with the corresponding outcomes presented in Figure 5.

The analysis results show that compared with base asphalt, the weight-average molecular weight (M_w_) and number-average molecular weight (*M_n_*) of aged asphalt increase significantly, and the polydispersity index (PDI) increases. This phenomenon indicates that polymerization or condensation reactions occur in asphalt molecules during aging [28], leading to the aggregation of large-molecular-weight asphaltenes and a subsequent rise in their relative proportion within the system, thus broadening the molecular weight distribution. This molecular change leads to the decrease in stability and performance deterioration of the aged asphalt system [29]. After regeneration, the *M_n_* and M_w_ of recycled asphalt decrease to varying degrees, and the PDI distribution narrows, which signifies that rejuvenators can efficiently lower the total content of macromolecular components in the system and reinstate the performance of aged asphalt back to that of the virgin asphalt.

Further comparative analysis shows that the action effects of the two rejuvenators present significant differences. The M_n_ and M_w_ of recycled asphalt A are closer to those of base asphalt, and the PDI is lower than that of recycled asphalt B. This suggests that Rejuvenator A provides more precise and effective control over the molecular weight distribution of aged asphalt, resulting in a narrower molecular distribution and higher structural homogeneity.

### 3.4. Analysis of Molecular Structure Evolution

**Figure 6:**
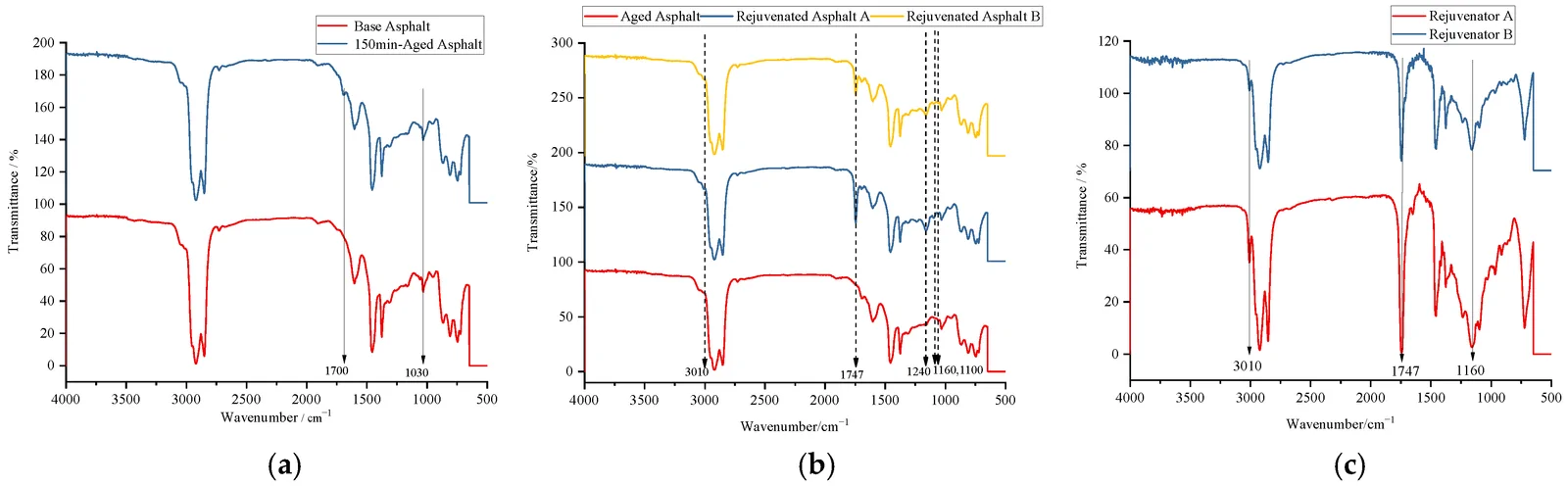
FTIR spectra. (**a**) Aged asphalt. (**b**) Rejuvenated asphalt. (**c**) Rejuvenators.

To reveal the essence of asphalt aging and rejuvenation at the molecular structure level, this study systematically compared the FTIR spectra of base asphalt [30], aged asphalt, rejuvenated asphalts, and the two rejuvenators, as shown in Figure 6 and Table 8.

Figure 6a and Table 8 show that after 150 min of RTFOT aging, the asphalt exhibited a characteristic carbonyl (C=O) peak for oxidative aging at 1710 cm^−1^, and the sulfoxide (S=O) peak at 1030 cm^−1^ intensified. Simultaneously, the aromatic C-H peak at 3050 cm^−1^ weakened, while the aliphatic C-H peaks at 2923 and 2850 cm^−1^ intensified [31].

The rejuvenation process significantly intervened in the above structural changes. As shown in Figure 6b, the rejuvenated asphalts displayed characteristic peaks of the rejuvenators at 3010 cm^−1^ (aromatic C-H), 1747 cm^−1^ (ester C=O), and 1240–1100 cm^−1^ (C-O-C) [31]. Meanwhile, the intensities of the aging characteristic peaks (carbonyl, sulfoxide) diminished, indicating that the rejuvenators effectively supplemented specific functional groups and reduced the relative concentration of oxidation products. Notably, the intensities of these newly appeared characteristic peaks in rejuvenated asphalt B were weaker than those in rejuvenated asphalt A, suggesting that it supplemented fewer aromatics and polar functional groups. This corroborates the SARA fraction analysis results and explains, from a structural perspective, the reasons for its relatively insufficient rejuvenation efficiency and compatibility.

The calculated (A_1710_ + A_1030_)/A_1457_ and A_1606_/A_720_ ratios [32] further confirm the chemical evolution during aging and regeneration. As shown in Table 8, the polarity index (A_1710_ + A_1030_)/A_1457_ increased from 0.043 in the base asphalt to 0.082 after 150 min of aging, reflecting the accumulation of carbonyl and sulfoxide groups during thermal-oxidative aging. This is consistent with the enhanced absorption at 1700 cm^−1^ and 1030 cm^−1^ observed in Figure 6a. Meanwhile, the aromaticity index A_1606_/A_720_ increased from 6.423 to 7.534, indicating the relative enrichment of aromatic structures and the increased condensation of asphaltenes during aging. This observation is in good agreement with the SARA analysis (Table 7), which showed that the asphaltene content increased from 9.57% to 15.91%. After rejuvenation, both indices decreased, with Rejuvenator A showing a more pronounced reduction than Rejuvenator B, consistent with its superior capacity to disperse asphaltene aggregates products.

Figure 6c analyzes the structure of rejuvenators and further reveals the root cause of the above differences. The absorption peak intensity of Rejuvenator A at 3010 cm^−1^, 1747 cm^−1^, and the C-O-C region is significantly higher than that of Rejuvenator B, proving its molecular structure characteristics of “high aromaticity” and “high polarity”. On the contrary, Rejuvenator B has a stronger aliphatic C-H stretching vibration peak at 2923 and 2850 cm^−1^, reflecting its “high aliphatic” structural characteristics. The above structural differences determine their differences in interfacial polarity, diffusion behavior, and interaction mode with aged asphalt. Notably, no new chemical bonds are detected during the whole regeneration process, confirming that the regeneration effect of both rejuvenators on aged asphalt is dominated by physical blending and strong physicochemical interactions rather than chemical reactions.

### 3.5. Molecular Dynamics (MD) Simulations of Rejuvenator–Aged Asphalt Systems

To provide molecular-level interpretation for the distinct regeneration behaviors of Rejuvenator A and Rejuvenator B observed in the preceding experiments, molecular dynamics (MD) simulation was performed on the rejuvenator–150 min-aged asphalt systems.

#### 3.5.1. Model Construction

**Figure 7:**
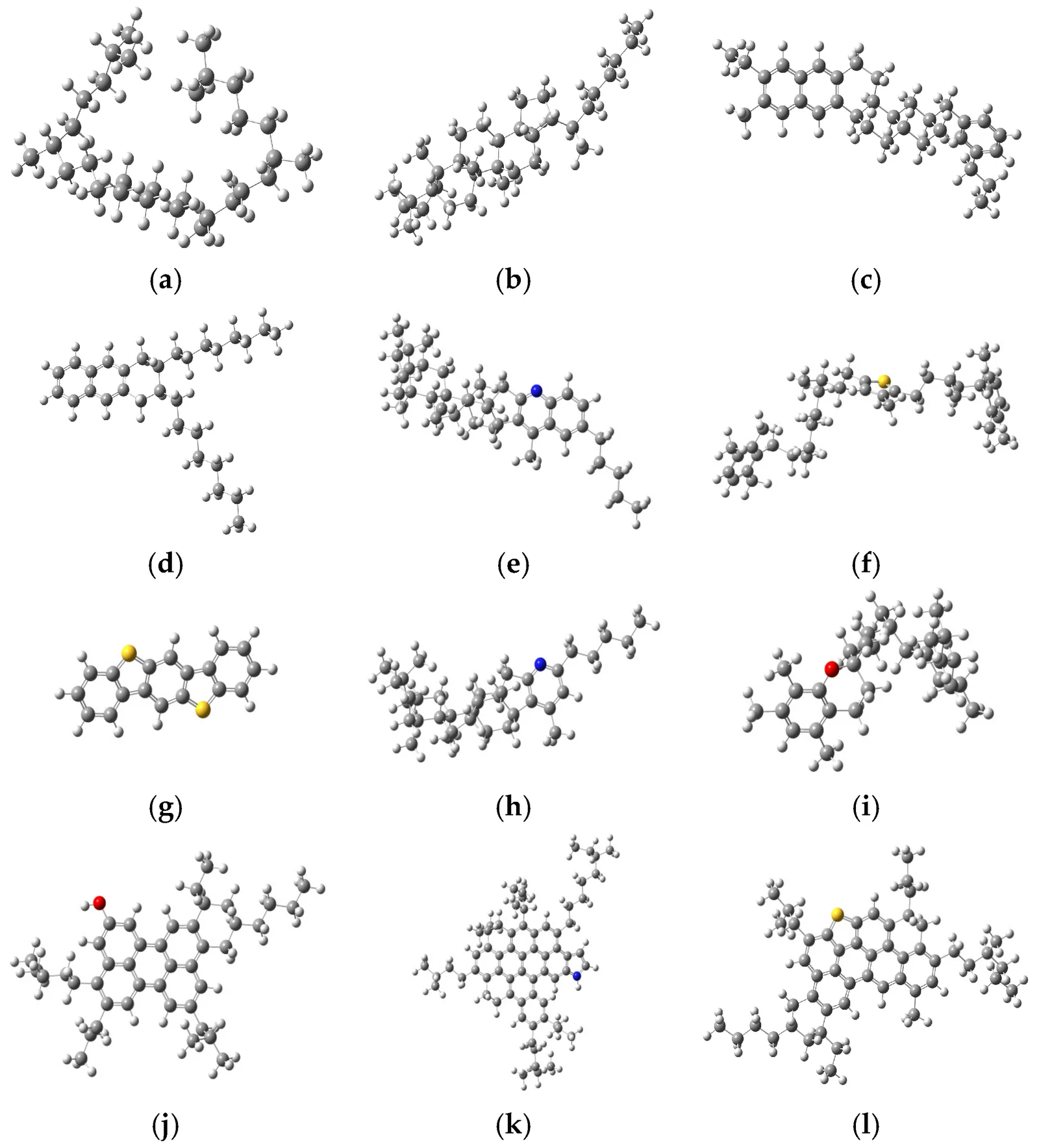
Molecular model of asphalt. (**a**) Saturates a. (**b**) Saturates b. (**c**) Aromatics a. (**d**) Aromatics b. (**e**) Resins a. (**f**) Resins b. (**g**) Resins c. (**h**) Resins d. (**i**) Resins e. (**j**) Asphaltenes a. (**k**) Asphaltenes b. (**l**) Asphaltenes c. Atom colors: dark-gray for carbon, light-gray for hydrogen, red for oxygen, blue for nitrogen, yellow for sulfur.

The aged asphalt molecular models were established based on the SARA fractionation results (Section 3.3.1) and previously reported asphalt molecular structures, consisting of representative molecules for asphaltenes (3 types), saturates (2 types), aromatics (2 types), and resins (5 types) [33], as shown in Figure 7. It should be noted that although multiple representative molecules are adopted for each SARA fraction, real asphalt contains thousands of diverse organic compounds. This simplified molecular combination cannot fully replicate all components of practical asphalt. Accordingly, subsequent analysis focuses on the relative variation trends of molecular interaction parameters rather than overinterpreting absolute energy values.

**Figure 8:**
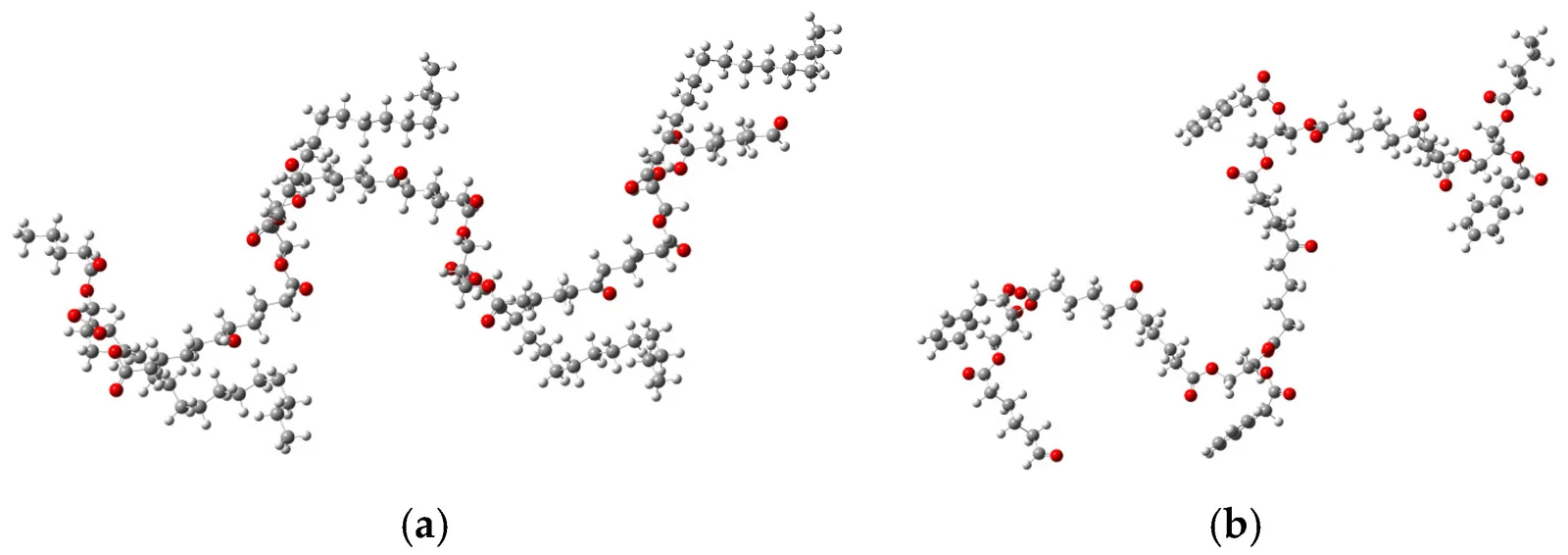
Molecular models of rejuvenators. (**a**) Rejuvenator A. (**b**) Rejuvenator B. Atom colors are consistent with Figure 7.

For the rejuvenators, as shown by the SARA analysis (Table 7), Rejuvenator A is dominated by aromatics (68.10%) and resins (20.17%) and contains abundant polar functional groups (ester C=O, ether C–O–C) as identified by FTIR (Section 3.4). Therefore, its representative molecule was constructed to embody these features: a relatively large aromatic core with ester/ether polar groups. Conversely, Rejuvenator B is characterized by a high saturates content (31.64%) and predominantly aliphatic structures with negligible polarity. Its representative molecule was accordingly built as a long-chain alkane to reflect its chemical nature and the absence of polar functional groups (as shown in Figure 8). Thus, while the simulated rejuvenator molecules are simplified representations, they are designed to capture the chemically distinctive features—polarity, aromaticity, and functional group types—that govern the interfacial and colloidal interactions discussed throughout this study. This approach is consistent with the established MD simulation protocols for asphalt–rejuvenator systems [34].

#### 3.5.2. Intermolecular Interactions

**Figure 9:**
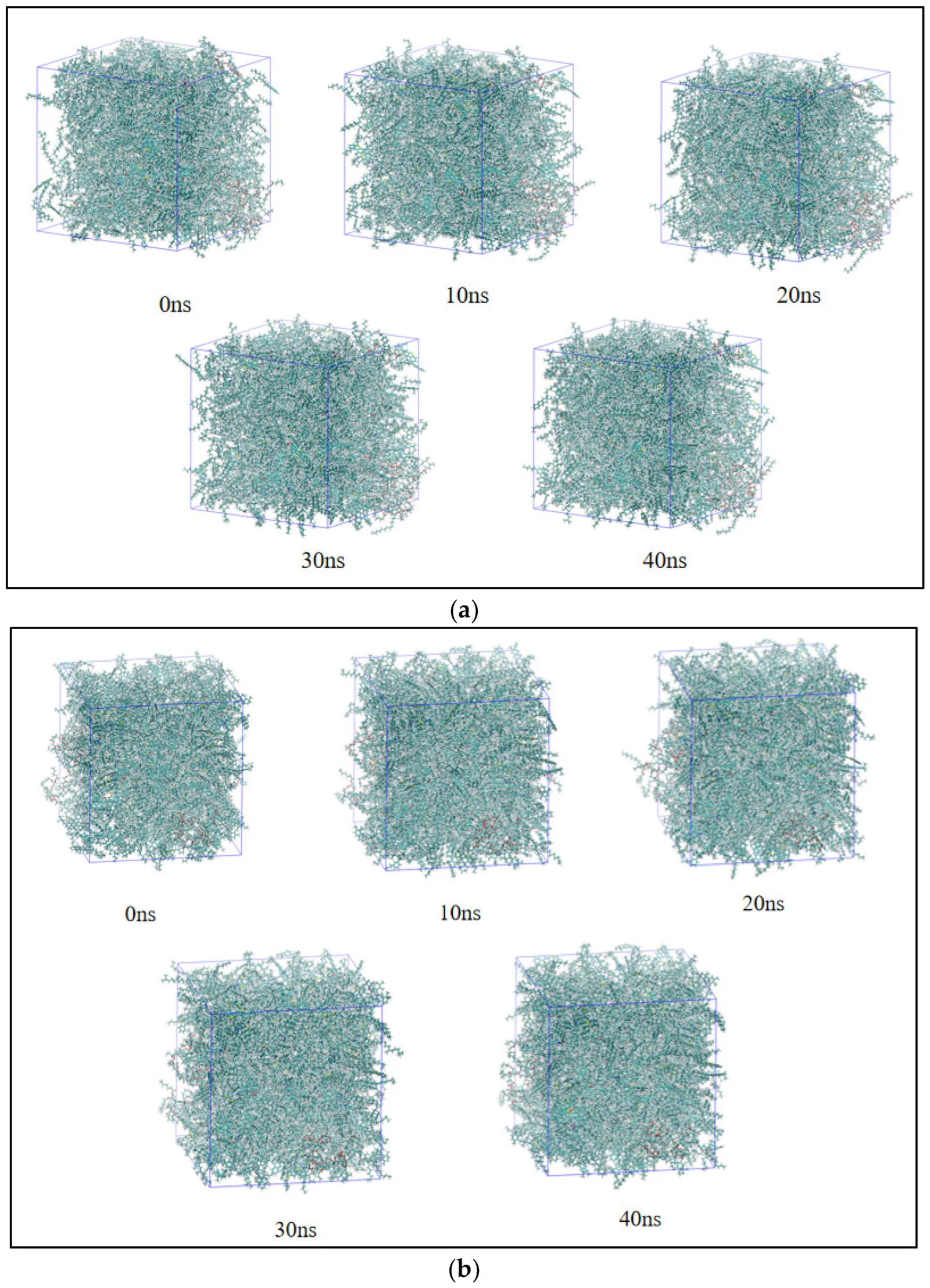
Molecular dynamics (MD) simulation processes of equilibrium phase (40 ns). (**a**) Equilibrium system of Rejuvenator A and aged asphalt. (**b**) Equilibrium system of Rejuvenator B and aged asphalt. In this figure, cyan-green spheres represent carbon atoms, and cyan-green lines denote carbon-carbon chemical bonds. Light-gray spheres for hydrogen, red for oxygen, blue for nitrogen, yellow for sulfur.

Figure 9 presents the equilibrium molecular configurations of the two simulated blended systems, revealing obvious differences in intermolecular interaction strength between the two blends.

**Figure 10:**
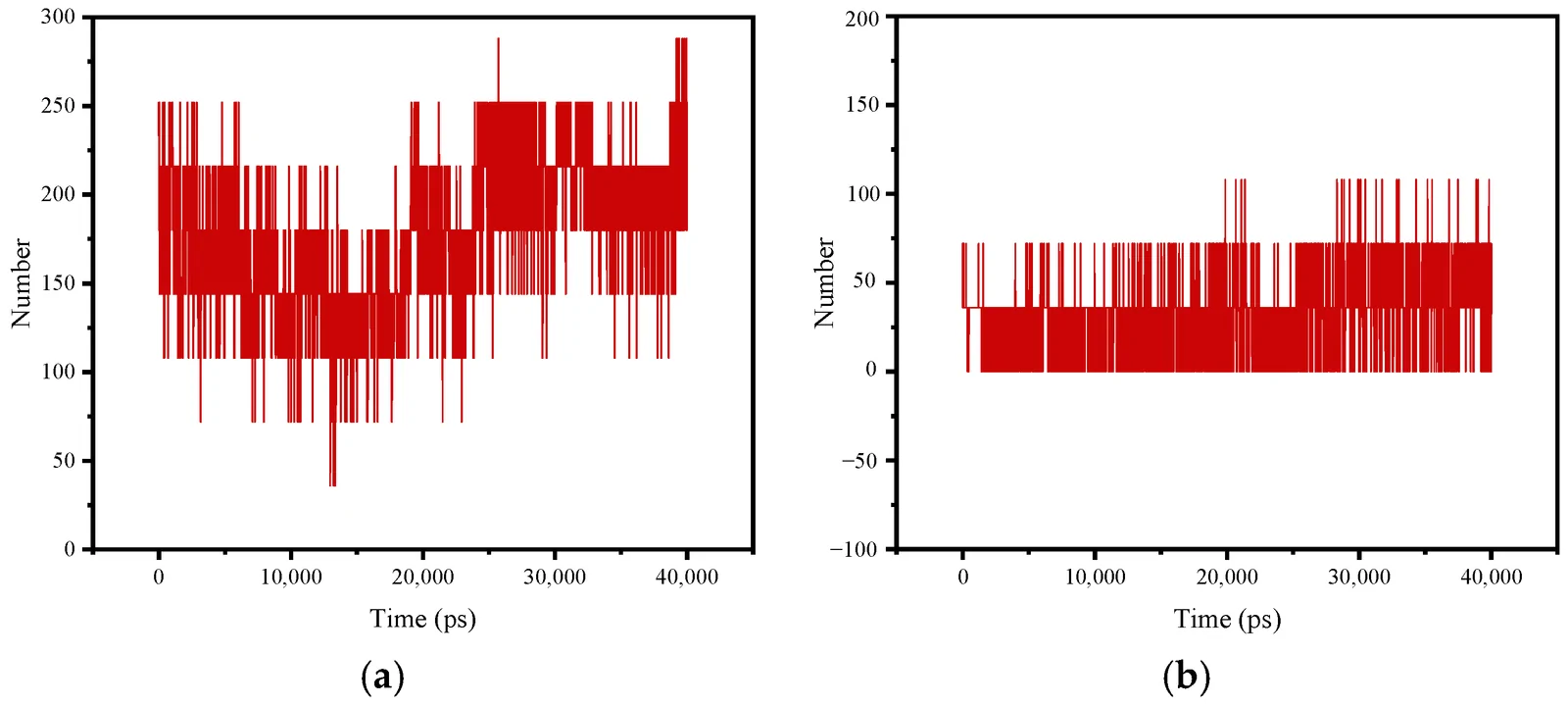
Count of hydrogen bonds within rejuvenator-aged asphalt systems. (**a**) Rejuvenator A and aged asphalt. (**b**) Rejuvenator B and aged asphalt.

The blended system of Rejuvenator A and aged asphalt yields 150–250 hydrogen-bond-like contacts under the geometric criteria, as shown in Figure 10. These interactions are mainly formed between oxygen-containing polar moieties of Rejuvenator A (ester C=O and ether C-O-C acting as hydrogen-bond acceptors) and hydroxyl-bearing groups (-OH) from aged asphalt components serving as hydrogen-bond donors. For the rejuvenator B system, 0–80 hydrogen-bond-like contacts are observed, which comprise both classical hydrogen bonds and weak C-H···O interactions. This is because Rejuvenator B possesses few polar groups capable of acting as conventional hydrogen-bond acceptors, resulting in a scarcity of classical hydrogen bonds and leaving weak C-H···O interactions as the primary contacts. The markedly larger number of contacts in the rejuvenator A system reflects prominent hydrogen-bond-like interactions. In contrast, the low number of contacts for the rejuvenator B system aligns well with its more pronounced aliphatic character compared to Rejuvenator A.

**Figure 11:**
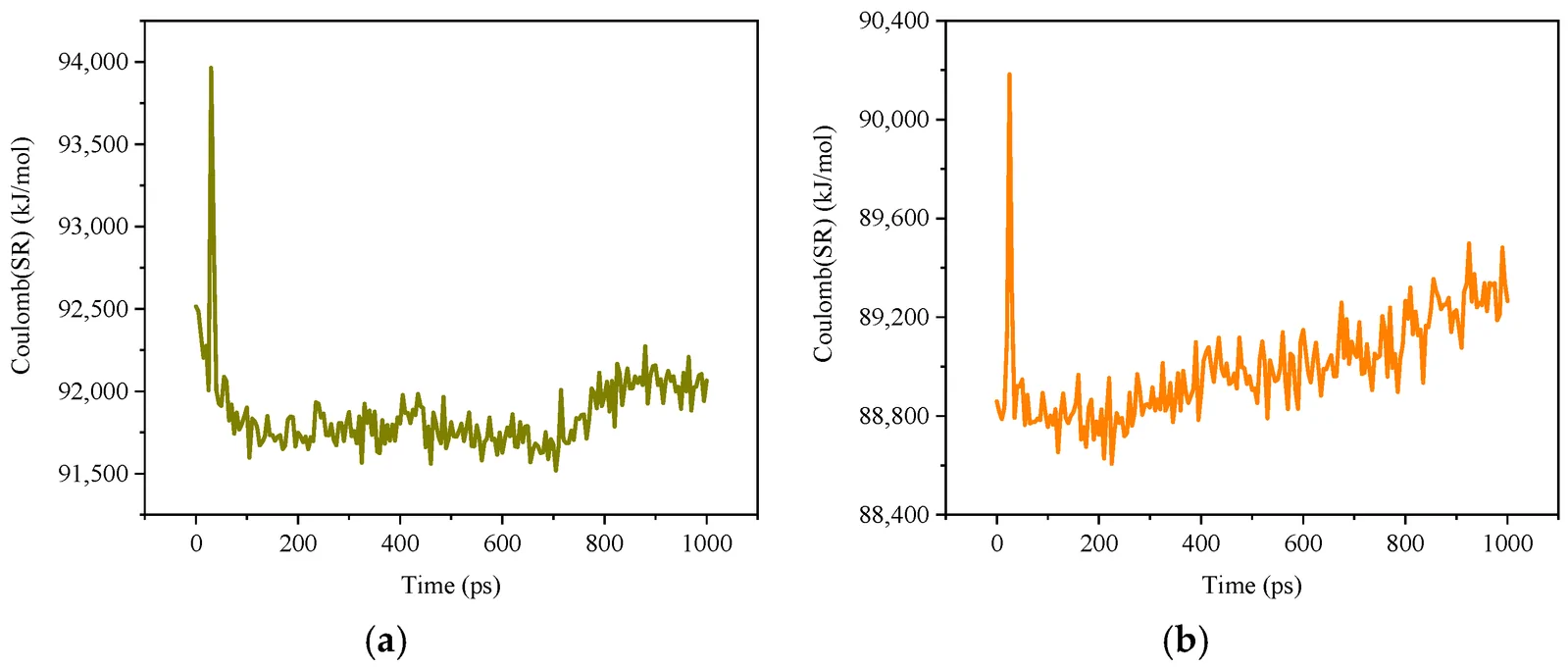
Variation in Coulomb potential energy of rejuvenator–aged asphalt systems with simulation time. (**a**) Variation in Coulomb potential of recycled asphalt A. (**b**) Variation in Coulomb potential of recycled asphalt B.

To further interpret the molecular source of such differences, the Coulomb potential energy was analyzed, as summarized in Figure 11. The system of aged asphalt blended with Rejuvenator A exhibits a Coulomb potential energy of −91,835 ± 161 kJ/mol, while the rejuvenator B blended system gives −89,030 ± 175 kJ/mol (values averaged over the equilibrium phase of 200–1000 ps). Although this energy difference (~2800 kJ/mol) accounts for only approximately 3% of the total Coulomb energy magnitude, it is statistically significant, as it far exceeds the standard deviation of the equilibrium fluctuations. This indicates that the observed trend is highly consistent with the molecular-structural features rather than simulation noise. Rejuvenator A carries abundant polar groups and can generate strong electrostatic interactions with oxidized asphalt fractions. In contrast, Rejuvenator B interacts with aged asphalt predominantly through van der Waals forces, corresponding to its higher (less negative) Coulomb potential energy. Taken together, the discrepancies in hydrogen-bond-like contacts and van der Waals energies contribute to the overall divergence of intermolecular interactions, which further explains the distinct regeneration performance of the two rejuvenators.

#### 3.5.3. System Stability

**Figure 12:**
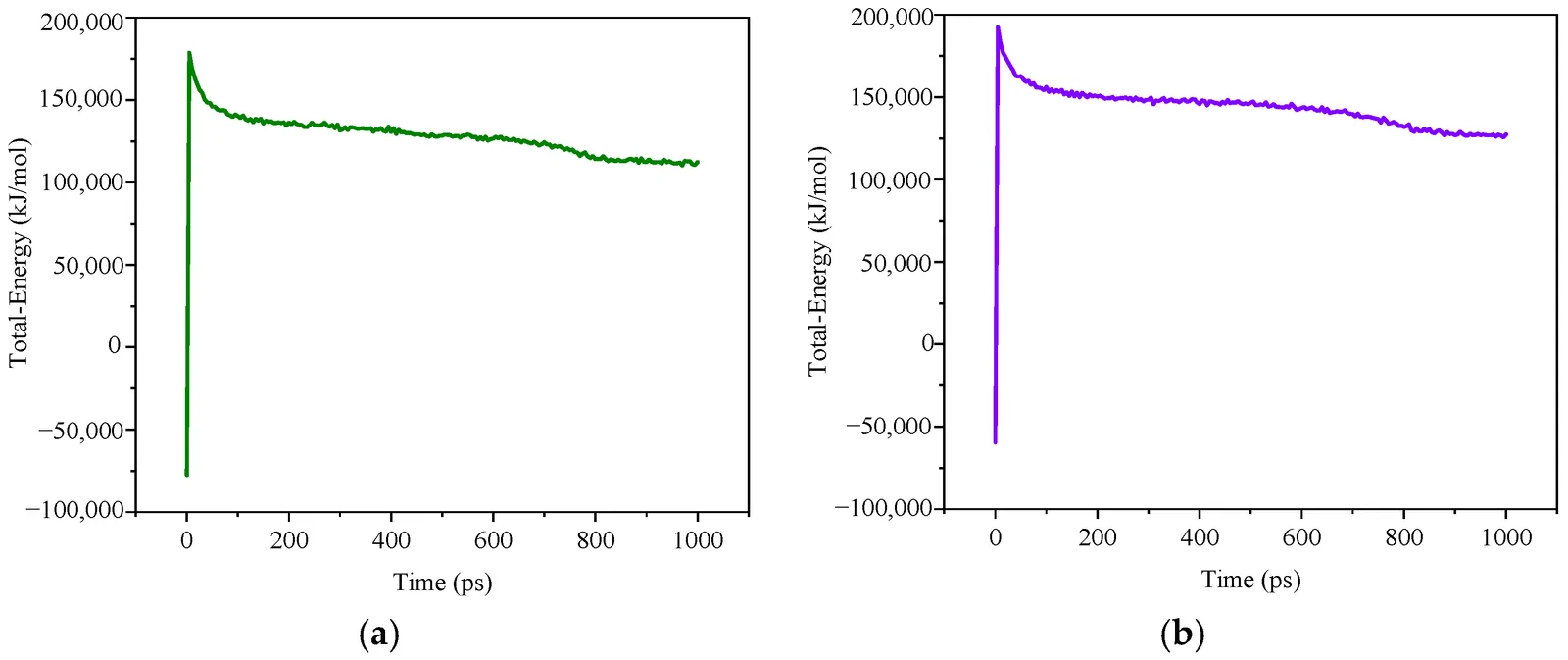
Variation in total energy of rejuvenator–aged asphalt systems with simulation time. (**a**) Rejuvenator A–aged asphalt system. (**b**) Rejuvenator B–aged asphalt system.

Figure 12 presents the total energy curves derived from molecular dynamics simulations. As illustrated, during the interaction between rejuvenators and aged asphalt, the total energy of both systems rises rapidly at first, then decreases progressively until reaching a balanced stable state. System A reaches a total energy peak of 179,000 kJ/mol and stabilizes at 110,000 kJ/mol, while System B exhibits a higher peak value of 192,000 kJ/mol with an equilibrium total energy of 127,000 kJ/mol. The higher equilibrium total energy of System B indicates looser molecular stacking within the asphalt compared with System A.

**Figure 13:**
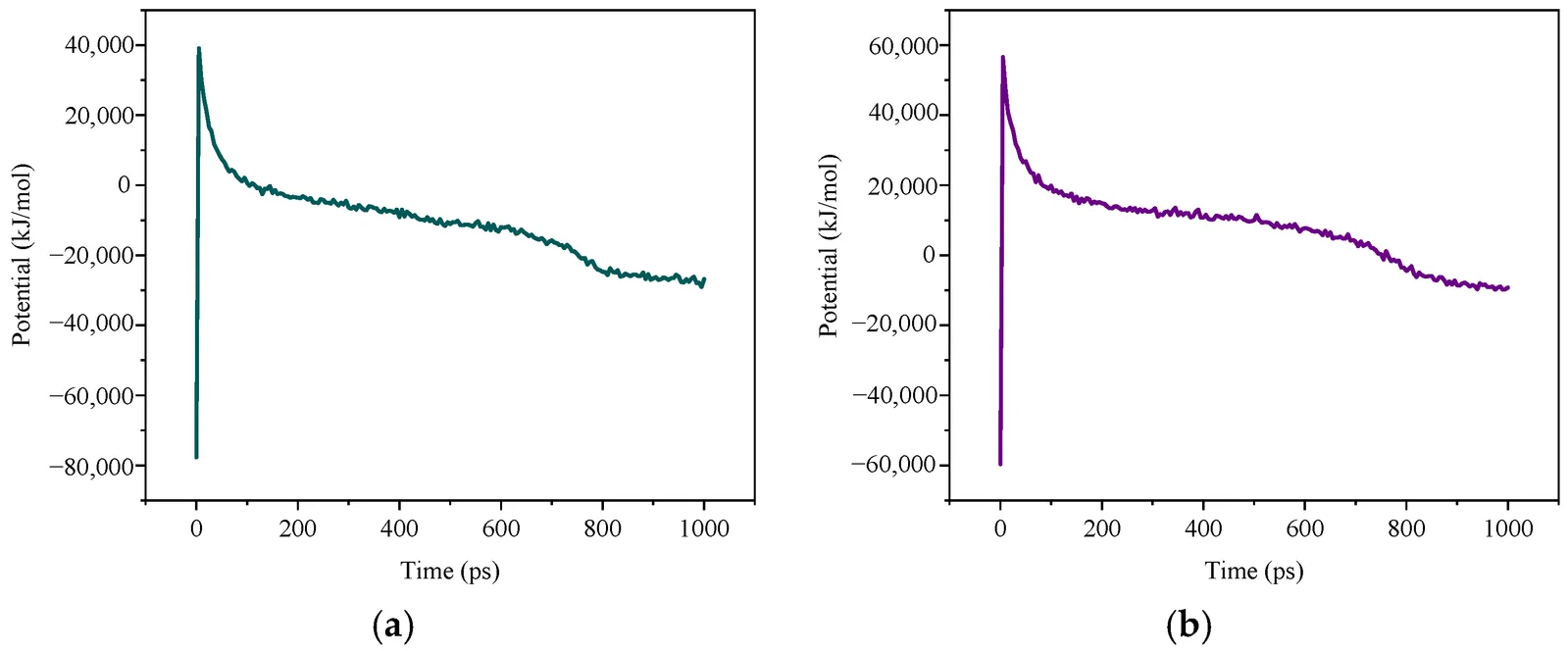
Variation in total potential energy of the systems with simulation time. (**a**) Rejuvenator A–aged asphalt system. (**b**) Rejuvenator B–aged asphalt system.

Figure 13 shows the potential energy variations of rejuvenated asphalt Systems A and B. The potential energy of System A stabilizes at −19,000 kJ/mol, whereas the equilibrium potential energy of System B is −9000 kJ/mol. As a fundamental thermodynamic parameter, the total energy of a system is the sum of its potential and kinetic energy. Calculated from the above data, System B exhibits a higher kinetic energy (136,000 kJ/mol) upon reaching equilibrium than System A (129,000 kJ/mol), which implies more violent molecular motion and stronger molecular fluidity within System B. Such microscopic dynamic characteristics directly determine macroscopic performance. Specifically, rejuvenated asphalt B has inferior structural stability and weaker deformation resistance, presenting fundamental performance differences compared with rejuvenated asphalt A.

## 4. Discussion

Based on the macro–meso–micro multi-scale test and simulation results above, this paper traces the root of performance differences between the two selected rejuvenators step by step from macroscopic apparent phenomena down to intermolecular interactions at the molecular scale.

### 4.1. Apparent Macroscopic Performance Gaps of Regenerated Asphalt

All test data in Section 3.1 proved that the two rejuvenators presented totally different recovery effects under gradient aging conditions. The selected Rejuvenator B restored the macroscopic indexes of 75 min-aged asphalt to the level of virgin binder, while the selected Rejuvenator A could well repair 112.5 min- and 150 min-aged asphalt. After secondary thermal aging, asphalt modified by Rejuvenator A maintained higher retention rates of penetration, softening point, and ductility. Such obvious macroscopic differences are the core phenomenon to be explained in the following multi-scale analysis.

### 4.2. Mesoscopic Interfacial Behavior Acts as the Superficial Cause of Performance Divergence

The interfacial test results in Section 3.2 can preliminarily interpret the above macroscopic gaps. The mesoscopic behaviors of the rejuvenator—ranging from initial contact and spreading to penetration—bridge its macroscopic performance and microscopic characteristics, serving as the critical link that dictates the final rejuvenating efficacy.

On the aged asphalt surface, Rejuvenator A exhibits a smaller contact angle and a higher spreading coefficient, resulting in a calculated relative penetration time roughly 13% shorter than that of Rejuvenator B. These superior mesoscopic properties facilitate rapid and uniform penetration into the aged asphalt matrix. This ensures sufficient microscopic interactions and component homogenization, serving as a critical prerequisite for its exceptional regeneration efficacy.

The diffusion process of Rejuvenator B is relatively slow, mainly due to its higher contact angle, leading to insufficient wetting and diffusion capacity. The rejuvenator has a slow apparent interfacial transfer rate and takes longer to achieve uniform mixing, which will limit the full play of its action in the actual short-time mixing process, and may further affect the uniformity of the regeneration system and the stability of final performance.

### 4.3. Micro-Component and Molecular Structural Internal Causes

The core of regeneration lies in restoring the asphalt colloid system imbalanced by aging. This study demonstrates that the two rejuvenators exhibit fundamentally different intervention mechanisms at this level.

Rejuvenator A functions primarily through component supplementation and the subsequent reorganization of the colloidal structure. As shown in Section 3.3.1, Rejuvenator A is rich in aromatics (68.10%) and resins (20.17%). During regeneration, high-concentration aromatics act as effective solvents to actively penetrate and dissolve asphaltene aggregates formed during aging, thus significantly reducing their concentration. Meanwhile, the large amount of supplemented resins provides a stable solvation layer for the redispersed asphaltene particles, and collaboratively reconstructs a stable colloidal structure. This mechanism ranging from component supplementation to structure reconstruction serves as the material basis for its deep regeneration.

Rejuvenator B mainly operates via a physical dilution-limited regulation mechanism, which relies on its high saturate content (31.64%). As low-polarity and low-viscosity components, saturates primarily function to reduce the average molecular weight and viscosity of the entire asphalt system, thereby exerting a dilution effect. Although this mechanism can lower the apparent concentration of asphaltenes to a certain extent, its supplementation of resin components is limited, as discussed in Section 3.3.1. Consequently, Rejuvenator B exhibits a weaker ability to reconstruct colloidal stability compared with Rejuvenator A. Its effect is primarily a physical regulation of the system’s rheological properties rather than a fundamental reconstruction of the colloidal structure.

### 4.4. Molecular Interactions and Structural Evolution

Evidence at the molecular level further refines and confirms the differences in the macroscopic compositional pathways described above.

Strong Polar Interactions of Rejuvenator A. As revealed by the FTIR analysis in Section 3.4, Rejuvenator A molecules possess a significantly higher content of polar oxygen-containing functional groups, including ester and ether groups. These strong polar groups can undergo robust interactions, and even hydrogen bonding, with the oxidized asphaltenes (rich in hydroxyl groups) in the aged asphalt. This strong interaction is not only the microscopic driving force for efficiently disrupting asphaltene aggregates but also ensures Rejuvenator A’s superior compatibility and interfacial bonding strength with the aged asphalt system, directly corresponding to its higher work of adhesion and better diffusion uniformity observed in Section 3.2.3.

Weak Polar Blending and Dissolution of Rejuvenator B. The FTIR spectrum of Rejuvenator B shows stronger aliphatic characteristics, with relatively fewer polar functional groups. Therefore, its interaction with aged asphalt relies more on van der Waals forces and blending dissolution in non-polar or weak polar environments. Its ability to “disassemble” the polar aggregates of aged asphalt is relatively weaker, and the strength and specificity of the interaction are insufficient, consistent with its relatively lower interfacial work of adhesion and diffusion efficiency.

### 4.5. Molecular Dynamics Reveals the Essential Polar Interaction Mechanism

The MD simulation results corroborate the experimental findings in three aspects.

First, the abundant hydrogen bonds (150–250) in the Rejuvenator A system provide molecular-scale evidence for the FTIR-observed polar interactions (Section 3.4), explaining why Rejuvenator A can actively dissolve asphaltene aggregates and reconstruct the colloidal structure (Table 7). This is further supported by the more negative Coulomb potential energy of the Rejuvenator A system.

Second, the low hydrogen bond count (0–80) and higher kinetic energy in the Rejuvenator B system support the physical dilution mechanism inferred from the SARA analysis (Section 3.3.1): Rejuvenator B, with a relatively high proportion of saturates, mainly reduces viscosity without actively disrupting asphaltene aggregates.

Third, the stronger intermolecular interactions in the Rejuvenator A system (higher Coulomb potential energy) are consistent with its superior interfacial bonding and diffusion behavior observed in Section 3.2. These simulations thus provide molecular-scale evidence that the distinct chemical compositions of the two rejuvenators govern their fundamentally different regeneration pathways: active polar interaction-driven reconstruction for Rejuvenator A versus passive physical dilution for Rejuvenator B.

It should be noted that macroscopic evaluation in this study mainly relies on conventional physical indices including penetration, softening point, and ductility. These parameters can intuitively reflect asphalt property changes induced by aging and rejuvenation and support multi-scale mechanism analysis. Nevertheless, systematic rheological characterizations represented by DSR, BBR, and MSCR are not fully covered. Future research will conduct these representative tests together with additional macroscopic characterization methods, aiming to build quantitative links between binder performance and micro-molecular mechanisms.

The quantitative findings are sample-specific, but the established multi-scale framework can guide the screening of rejuvenators for RAP applications. Future research will select multiple rejuvenators with varied aromatic and polar-group contents for comparative tests, to further derive general component-design principles for asphalt rejuvenators.

Further validation under field conditions and at the mixture scale is recommended.

## 5. Conclusions

This study hypothesized that compositional differences between bio-based Rejuvenator A and petroleum-based Rejuvenator B would lead to distinct regeneration effects on asphalt aged for 75, 112.5, and 150 min. A multi-scale framework combining asphalt binder tests, interfacial characterization, SARA fractionation and FTIR spectroscopy, and COMPASS II MD simulations was built to verify this hypothesis, and enabled comparative mechanistic analysis. Key quantitative conclusions are listed below:

(1) Rejuvenator B restored 75 min-aged asphalt mainly through physical dilution, whereas Rejuvenator A effectively recovered 112.5 min- and 150 min-aged asphalt, and the rejuvenated binder exhibited lower oxidative aging than that restored by Rejuvenator B.
(2) Mesoscopically, Rejuvenator A exhibited superior diffusion performance driven by favorable interfacial energy compatibility with aged asphalt. This resulted in a lower equilibrium contact angle and enhanced spreading, with a relative penetration time roughly 13% shorter than that of Rejuvenator B.
(3) Chemically, Rejuvenator A contained high aromatics (68.10%) and polar functional groups, which physically disrupted asphaltene agglomerates and reconstructed colloidal structures. The colloidal instability index (CII) decreased from 0.427 (aged asphalt) to 0.180 for rejuvenated asphalt A, substantially lower than the 0.293 for rejuvenated asphalt B.
(4) MD simulations confirmed that Rejuvenator A formed 150–250 hydrogen bonds with aged asphalt, compared to only 0–80 for Rejuvenator B, with a more negative Coulomb potential energy. These strong electrostatic interactions drove the structural reorganization of Rejuvenator A, whereas Rejuvenator B relied mainly on weak van der Waals forces.
(5) The superior performance of Rejuvenator A is governed by its chemical composition—high aromatics, high resins, and polar functional groups—rather than its bio-based origin. These compositional features provide a rationale for designing high-performance rejuvenators by tailoring molecular structures, regardless of feedstock source.

## Figures and Tables

**Table 1:** Technical specifications of 90# base asphalt.

Item	Specification	Test Result	Test Method
Penetration (25 °C, 5 s, 100 g)/0.1 mm	80–100	86.1	T 0604
Softening Point (Ring-and-Ball Method)/°C	≥44	46.5	T 0606
Ductility (15 °C)/cm	≥100	>100	T 0605
Dynamic Viscosity at 60 °C/Pa·s	≥140	188.8	T 0625

**Table 2:** Fundamental properties of rejuvenators.

Item	Rejuvenator A	Rejuvenator B	Test Method
Kinematic Viscosity at 60 °C/mm^2^/s	42.8	45.2	T 0619
Flash Point/°C	235	228	T 0611
Density (15 °C)/g·cm^−3^	0.9062	0.9056	T 0603

**Table 3:** Main equipment and instruments.

Equipment Name	Model	Manufacturer
Rotational Viscometer	NDJ-1C	Xianxian Yaxing Highway Construction Instrument Factory, Cangzhou, China
Infrared Spectrometer	Nicolet iS 5 FTIR	ThermoFisher Scientific, Waltham, MA, USA
Contact Angle Goniometer	CA200	Guangdong Beidou Precision Instrument Co., Ltd., Dongguan, China
Thin-layer Chromatograph	SF-16A	Beijing Jiahang Bochuang Technology Co., Ltd., Beijing, China
Gel Permeation Chromatograph	Waters 515-717-2410	Waters Corporation, Milford, MA, USA

**Table 4:** Diffusion calculation results for rejuvenators.

Rejuvenator	Surface Tension (mN/m)	Kinematic Viscosity (mm^2^/s)	Substrate Sample Name	Contact Angle (°)	Relative Interfacial Transfer Rate	Relative Transfer Time
A	34.84	42.8	Base asphalt	20.811	0.190	2.629
A	34.84	42.8	75 min	30.645	0.175	2.856
A	34.84	42.8	112.5 min	34.171	0.168	2.972
A	34.84	42.8	150 min	36.595	0.163	3.060
B	33.12	45.2	Base asphalt	25.593	0.165	3.026
B	33.12	45.2	75 min	34.858	0.150	3.331
B	33.12	45.2	112.5 min	37.185	0.146	3.429
B	33.12	45.2	150 min	39.171	0.142	3.519

**Table 5:** Interaction results between rejuvenators and asphalt.

Rejuvenator	γ*_LV_* (mN/m)	Substrate Sample Name	θ(°)	*W*_a_ (mN/m)	*S* (mN/m)	*W*_i_ (mN/m)
A	34.84	Base asphalt	20.811	67.40	−2.27	32.56
A	34.84	75 min	30.645	64.82	−4.85	29.97
A	34.84	112.5 min	34.171	63.65	−6.01	28.82
A	34.84	150 min	36.595	62.81	−6.86	27.97
B	33.12	Base asphalt	25.593	62.99	−3.25	29.86
B	33.12	75 min	34.858	60.29	−5.94	27.18
B	33.12	112.5 min	37.185	59.50	−6.73	26.38
B	33.12	150 min	39.171	58.78	−7.46	25.66

**Table 6:** Softening point results from segregation tests of rejuvenated asphalts.

Rejuvenated Asphalt Type	Top Softening Point/°C	Bottom Softening Point/°C	Softening Point Difference/°C
Rejuvenated Asphalt A	43.2	43.3	0.1
Rejuvenated Asphalt B	47.8	48.2	0.4

**Table 7:** SARA analysis results.

SARA Fraction/%	Base Asphalt	Aged Asphalt	Rejuvenated Asphalt A	Rejuvenated Asphalt B	Rejuvenator A	Rejuvenator B
Asphaltene	9.57	15.91	7.04	7.84	1.42	1.28
Resin	29.75	29.80	38.86	30.52	20.17	15.05
Aromatic	44.90	40.27	45.92	46.80	68.10	52.03
Saturate	15.78	14.03	8.18	14.84	10.31	31.64
CII	0.340	0.427	0.180	0.293	-	-

**Table 8:** Integrated absorption areas of characteristic FTIR bands and calculated structural indices of asphalt samples.

Characteristic Peaks /cm^−1^				Peak Area A	
		Base Asphalt	150 min-Aged Asphalt	Rejuvenated Asphalt A	Rejuvenated Asphalt B
3200–3500		39.657	119.196	0	0
3050		0.390	0.368	0.257	0.267
1606		8.953	8.491	5.351	5.478
900~650	865	6.601	6.126	5.008	5.002
	810	5.141	4.633	4.289	4.468
	743	3.841	3.220	3.034	3.338
2923		54.845	55.994	58.385	58.878
2850		16.490	17.410	17.400	17.590
1457		29.753	30.269	28.392	28.922
1377		5.585	5.438	5.609	5.543
720		1.394	1.127	1.313	1.196
966		0.820	0.518	0.669	0.582
1030		1.270	1.663	1.372	1.582
1710		0	0.808	0.463	0.549
3010		0	0	0.223	0.092
1747		0	0	6.671	2.401
1160		1.329	1.364	5.966	2.470
1240		0	0	0.402	0.183
1100		0.024	0.011	0.644	0.272
(A_1710_ + A_1030_)/A_1457_		0.043	0.082	0.065	0.074
A_1606_/A_720_		6.423	7.534	4.075	4.580

## Data Availability

The original contributions presented in this study are included in the article. Further inquiries can be directed to the corresponding author.

## References

[1] Decký M., Remišová E., Samek M. (2025). Research on the possibilities of reusing mixed reclaimed asphalt materials with a focus on the circular economy. Appl. Sci..

[2] Aitkaliyeva G., Ashimova S., Baidullayev I., Merkibayev Y., Yelubay M., Toleutay G., Rossi C.O. (2025). Oil sludge as a rejuvenator for aged bitumen: Structural and thermal effect. Appl. Sci..

[3] Sagar K.M., Venudharan V., Saha G. (2026). Application of bio-rejuvenators to maximize the performance and durability of reclaimed asphalt pavement incorporated mixtures: State-of-the-art review. Innov. Infrastruct. Solut..

[4] Singhal P., Gupta A.B. (2024). Application of waste cooking oil as a rejuvenator for aged bitumen. IOP Conf. Ser. Earth Environ. Sci..

[5] Tabaković A., van Vliet D., Roetert-Steenbruggen K., Leegwater G. (2023). Bio-oils as asphalt bitumen rejuvenators. Eng. Proc..

[6] Camargo I., Hofko B., Graziani A., Grilli V. (2023). Dilauryl thiodipropionate as a regeneration agent for reclaimed asphalts. Constr. Build. Mater..

[7] Al-Saffar Z.H., Hasan H.G.M., Mohialdeen O.K., Dulaimi A. (2024). Assessing the effectiveness of bio-based oil in rejuvenating aged asphalt: A comprehensive physical, rheological, chemical, and mechanical examination. Innov. Infrastruct. Solut..

[8] Wang R., Chen X., Cheng P. (2026). Review on the mechanism and performance enhancement of biomass-based rejuvenators in reclaimed asphalt recycling. Polymers.

[9] Cavalli M.C., Wu W., Poulikakos L. (2024). Bio-based rejuvenators in asphalt pavements: A comprehensive review and analytical study. J. Road Eng..

[10] Li D.D., Greenfield M.L. (2014). Chemical compositions of improved model asphalt systems for molecular simulations. Fuel.

[11] Zhang L., Greenfield M.L. (2007). Analyzing properties of model asphalts using molecular simulation. Energy Fuels.

[12] Sun H. (1998). COMPASS: An ab initio force-field optimized for condensed-phase applications. J. Phys. Chem. B.

[13] (2004). Technical Specifications for Construction of Highway Asphalt Pavements.

[14] (2019). Technical Specifications for Highway Asphalt Pavement Recycling.

[15] (2025). Standard Test Methods of Asphalt and Asphalt Mixture for Highway Engineering.

[16] Li Y., Hui B., Yang X., Wang H., Xu N., Feng P., Ma Z., Wang H. (2021). Multi-Scale Characterization of High-Temperature Properties and Thermal Storage Stability Performance of Discarded-Mask-Modified Asphalt. Materials.

[17] Gan X.L., Zhang W.L., Li J., Tang H. (2022). Research on the effect of SBS modifier content on the adhesion between asphalt and aggregate based on surface energy theory. Highway.

[18] Yaseen G., Hafeez I. (2020). Effect of Cereclor as Rejuvenator to Enhance the Aging Resistance of Reclaimed Asphalt Pavement Binder. Materials.

[19] Zhang W.X., Li Q., Wang J.Q. (2022). Aging behavior of high-viscosity modified asphalt binder based on infrared spectrum test. Materials.

[20] Wang Z., Pei Q., Li K., Wang Z., Huo X., Wang Y., Zhang X., Kong S. (2024). Molecular Dynamics Simulation of the Rejuvenation Performance of Waste Cooking Oil on Aged Asphalt. Molecules.

[21] Ji H.D., Li B., Yao T.F., Liu Z., Han J., Li A. (2023). Polyurethane and nano-TiO_2_ modifiers mitigate aging of asphalt binders by inhibiting aggregation of polar molecules: A molecular dynamics study. Colloids Surf. A Physicochem. Eng. Asp..

[22] Leodarta H., El-Ashwah A.S., Rath P., Abdelrahman M., Buttlar W.G. (2025). Utilization of FTIR-ATR for material characterization and forensic analysis. Constr. Build. Mater..

[23] Li Y., Li Z., Cao Y., Ding Z., Chang C., Weng Y., Hu C. (2026). Regeneration performance and mechanism of aged SBS high-viscosity modified bitumen: Influence of rejuvenator components and recycling strategies. Constr. Build. Mater..

[24] Cao Z., Huang X., Yu J., Han X., Wang R., Li Y. (2021). Laboratory evaluation of the effect of rejuvenators on the interface performance of rejuvenated SBS modified bitumen mixture by surface free energy method. Constr. Build. Mater..

[25] Dintcheva T.N., Teresi R., Graziano F., Infurna G., Volpe M., Messineo A., Celauro C. (2026). Impact of waste-hydrochar on the rheological behavior, physical properties, and aging resistance of bitumen. Materials.

[26] Lesueur D. (2009). The colloidal structure of bitumen: Consequences on the rheology and on the mechanisms of bitumen modification. Adv. Colloid Interface Sci..

[27] Zhang X., Han C., Zhou X., Otto F., Zhang F. (2021). Characterizing the diffusion and rheological properties of aged asphalt binder rejuvenated with bio-oil based on molecular dynamic simulations and laboratory experimentations. Molecules.

[28] Liu G., Nielsen E., Komacka J. (2014). Rheological and chemical evaluation on the ageing properties of SBS polymer modified bitumen: From the laboratory to the field. Constr. Build. Mater..

[29] Chen M.Z., Yu J.J., Zhang J.W., Leng B., Ma J. (2025). Mechanism of low-molecular-weight polyolefin in improving bitumen-aggregate interfacial adhesion. Chin. J. Process Eng..

[30] Muñoz C.S.J., Airey G. (2025). Ageing impact of hydrated lime in bitumen-filler mastics using infrared spectroscopy. Constr. Build. Mater..

[31] Perfler L., Cuk B., Chankov G., Traxl R., Hirzinger P., Lackner R. (2025). Effect of different rejuvenators and aging methods on the physicochemical properties of bitumen assessed by infrared spectroscopy and rheological analysis. Constr. Build. Mater..

[32] Yadykova A.Y., Strelets L.A., Ilyin S.O. (2023). Infrared Spectral Classification of Natural Bitumens for Their Rheological and Thermophysical Characterization. Molecules.

[33] Liang C., Liu Z., Sun C., Ye S., Yang X., Li Q., Du P., Zhang H. (2025). Investigation on the rejuvenation mechanism and molecular diffusion behavior of SBS modified asphalt rejuvenated from colza oil based on molecular dynamics simulation. Constr. Build. Mater..

[34] Wang R., Zhao Y., Sun Q. (2025). A molecular insight into asphalt-aggregate interfacial debonding: The role of hydrogen bonds in water molecule migration. Appl. Surf. Sci..

